# Superstructure-Based Optimization of Vapor Compression-Absorption Cascade Refrigeration Systems

**DOI:** 10.3390/e22040428

**Published:** 2020-04-10

**Authors:** Sergio F. Mussati, Tatiana Morosuk, Miguel C. Mussati

**Affiliations:** 1 INGAR Instituto de Desarrollo y Diseño (CONICET–UTN), Avellaneda 3657, S3002GJC Santa Fe, Argentina; mussati@santafe-conicet.gov.ar; 2 Institute for Energy Engineering, Technische Universität Berlin, Marchstr. 18, 10587 Berlin, Germany; tetyana.morozyuk@tu-berlin.de

**Keywords:** combined refrigeration process, absorption-compression, cascade, R134a (1,1,1,2-tetrafluoroetano), water-lithium bromide, double-effect, superstructure, optimization

## Abstract

A system that combines a vapor compression refrigeration system (VCRS) with a vapor absorption refrigeration system (VARS) merges the advantages of both processes, resulting in a more cost-effective system. In such a cascade system, the electrical power for VCRS and the heat energy for VARS can be significantly reduced, resulting in a coefficient of performance (COP) value higher than the value of each system operating in standalone mode. A previously developed optimization model of a series flow double-effect H_2_O-LiBr VARS is extended to a superstructure-based optimization model to embed several possible configurations. This model is coupled to an R134a VCRS model. The problem consists in finding the optimal configuration of the cascade system and the sizes and operating conditions of all system components that minimize the total heat transfer area of the system, while satisfying given design specifications (evaporator temperature and refrigeration capacity of −17.0 °C and 50.0 kW, respectively), and using steam at 130 °C, by applying mathematical programming methods. The obtained configuration is different from those reported for combinations of double-effect H_2_O-LiBr VAR and VCR systems. The obtained optimal configuration is compared to the available data. The obtained total heat transfer area is around 7.3% smaller than that of the reference case.

## 1. Introduction

Refrigeration is one of the varieties of low-temperature thermal engineering applications in different industries such as large food and drink industries, refineries and chemical plants, mechanical engineering, electronic devices, and other types of industries. Currently, the refrigeration industry is playing an important and increasing role in the global economy [1]. Therefore, an intense research and development effort is still required in this area [2,3].

Vapor compression refrigeration systems (VCRS) are the most widely used commercially, followed by the vapor absorption refrigeration systems (VARS). The values of the coefficient of performance (COP) and sizes of the equipment of VCRSs are higher and smaller, respectively, than for VARSs. Additionally, VCRSs can obtain refrigeration temperatures lower than VARSs. The former can be applied for refrigeration temperatures within the operating range between 300 and 120 K while the latter within the range between 280 and 243 K. The high electrical power consumption for VCRSs is still the main drawback of these systems. Advantageously, not much electrical power is required for VARSs since only a small amount of energy to power a pump is necessary; consequently, they are preferred in places where electrical power is expensive or difficult to access. In addition, non-conventional or renewable sources of energy such as geothermal and solar energy, biofuels, and low-grade waste energy can be employed to run VARSs [4]. As there are no moving part inside of components, the maintenance cost of VARSs is relatively low. However, the COP value of VARSs is comparatively low and the investment cost is high, which are the main drawbacks associated with these systems. 

Significant research efforts on modeling, simulation, and optimization are currently being put towards overcoming these drawbacks. A literature review reveals that there are many publications addressing the simulation and optimization of double-effect VARSs employing different methodologies: exergy analysis [5,6,7], exergoeconomic analysis [8,9], evolutionary algorithms [5,10], and rigorous optimization algorithms [11,12]. Arshad et al. [5] optimized the operating conditions to maximize the exergy efficiency of series and parallel flow double-effect H_2_O-LiBr VARS configurations. The main optimization variables included the operating temperatures at the high and low-temperature generators, evaporator, condenser, and absorber. A pressurized heated water was used as a heating utility in the generators. The authors successfully applied an evolutionary algorithm (genetic algorithm) supported by MATLAB. For a cooling capacity of 300 kW, it was found that the exergy efficiency and COP values obtained for the parallel flow configuration are higher than those obtained for the series configuration. Garousi Farshi et al. [8] applied the exergoeconomic method to analyze in detail three configurations of double-effect H_2_O-LiBr VARSs for a cooling capacity of 1 kW, arranged in series, parallel, and reverse parallel flow patterns. Pressurized steam was used as a heating utility. The Engineering Equations Solver (EES) software was used to implement the models. For each configuration, the influence of the operating temperature at the high-temperature generator, condenser, absorber, and evaporator on the total investment cost was studied. The evaporator temperature varied from 277 to 283 K. For the three examined configurations, the authors determined that the absorber and evaporator are the most influential process units to the total cost. Mussati el al. [11] optimized the operating conditions and process-unit sizes of a double-effect H_2_O-LiBr VARS with a series flow configuration for a cooling capacity of 300 kW and evaporator temperature of 279 K, using saturated steam at 404 K as a heating utility. To this end, they developed a nonlinear mathematical programming (NLP) model, which was solved with a rigorous optimization algorithm based on the generalized reduced gradient (GRG) method. As a result, a novel configuration was obtained by minimizing the total annual cost (investment and operating costs). The obtained configuration differs from the conventional in that in the former the low-temperature solution heat exchanger is removed from the process.

Regarding VCRSs, recent results on modeling and optimization considering single-objective functions can be found in [13,14,15,16,17] and multi-objective functions in [18,19]. By using energy, exergy, and economic analyses, Baakeem et al. [14] theoretically investigated a multi-stage VCRS considering eight refrigerants (R407C, R22, R717, R134a, R1234yf, R1234ze(E), R410A, and R404A). The proposed model was implemented in the EES software and solved using the conjugate directions method. The superheating and subcooling degrees and the operating temperatures at the condenser and evaporator were considered as the optimization variables. For a cooling capacity of 1 kW and a temperature of 273 K at the evaporator, the refrigerant R717 showed the highest COP value; the refrigerant R407C was not suggested to use due to the low exergy efficiency and high operating cost. Zendehboudi et al. [18] investigated the performance of VCRSs operating with R450A for cooling capacity values ranging between 0.5 and 2.5 kW. To this end, they developed a multi-objective optimization (MOO) approach coupling the response surface method (RSM) with the non-dominated sorting genetic algorithm II (NSGA-II) method to perform simulation-based optimizations. A case study consisted in minimizing the electrical power required by the compressor and its discharge temperature and maximizing the refrigerant mass flow rate. A second case study consisted in maximizing the cooling capacity and the refrigerant mass flow rate and minimizing the discharge temperature. As in [14], the evaporator and condenser temperatures and the superheating and subcooling degrees were parametrically optimized. The evaporator temperature was varied between 258 and 288 K. A result indicated that the cooling capacity is strongly influenced by the evaporator temperature.

An integrated system, which combines a VCRS with a VARS, merges the advantages of both standalone systems, resulting in a cost-effective refrigeration system [20,21]. In a combined VCR-VAR system—CVCARS—the electrical power required in VCRS and the heat energy required in VARS can be significantly reduced. This leads to an increase in the COP value [22,23]. There are many publications addressing exergy and exergoeconomic analyses of combined VCR–VAR systems that use different mixtures for the absorption cycle (e.g., H_2_O-LiBr and NH_3_-H_2_O) and different working fluids for the compression cycle (e.g., R22, R134a, R717, and R1234yf) [20,23,24,25,26,27,28,29,30,31]. Agarwal et al. [20] analyzed an absorption-compression cascade refrigeration system (ACCRS) by combining a series flow triple-effect H_2_O-LiBr VARS with a single VCRS operated with R1234yf. High-pressure generator, evaporator, and absorber temperature values ranging between 448.15 and 473.15 K, 223.15 and 263.15 K, and 298.15–313.15 K, respectively, were considered. The authors applied exergy analysis to calculate the performance parameters. They found that the amount of energy recovered in this configuration allowed to drastically reduce the energy input in the high-temperature generator and, consequently, the operating cost. No significant increases in the process-unit sizes were observed. Colorado and Rivera [31] presented a theoretical study to compare, from the point of view of the first and second laws of thermodynamics, the integration of VCRS and VARS considering both single and double-stage configurations for VARS, and using CO_2_ and R134a for VCRS and H_2_O-LiBr for VARC. As a result, they found that the highest irreversibilities are in the absorber and evaporator for both mixtures. Additionally, they concluded that, independently of the configuration (single or double-stage arrangements) the total irreversibility obtained for R134a/H_2_O-LiBr is significantly lower than that obtained for CO_2_/H_2_O-LiBr.

Exergy and exergoeconomic analyses are valuable tools to more accurately identify and quantify the thermodynamic inefficiencies associated with the components and the overall system. However, the application of the exergy analysis in order to obtain the optimal process configuration may require excessive computation time and a large number of iterations when the process under study involves many pieces of equipment. In addition, the designer’s interpretation plays an important role in obtaining improved process designs [32]. Several authors have applied genetic algorithms (GAs) to optimize ACCRS [24,33]. By using the NSGA-II technique, Jain et al. [33] solved a MOO problem for a single-effect H_2_O-LiBr VARS coupled to a VCRS operated with R410A, for a specified cooling capacity of 170 kW. The objective function selected was the minimization of the total irreversibility rate (as a thermodynamic criterion) and the total product cost (as an economic criterion). The authors compared the solutions obtained for the MOO problem with those obtained by considering the individual single-objective functions, concluding that the former are preferred over the latter. GAs, a class of evolutionary algorithms, were successfully applied in optimizing not only refrigeration systems but also complex engineering problems. In GAs, only the values of the objective functions are used without requiring any information about the gradient of the function at the evaluated points. Depending on the cases, GAs can obtain solutions close to the optimal solution in reasonable computation time. However, GAs require many input parameters that can influence the obtained solutions. Recently reported experimental results on CVCARSs can be found in [34,35].

Based on the available information, it can be concluded that different combinations of vapor compression and vapor absorption processes are promising options for combined refrigeration systems; therefore, there is a need to continue investigating and assessing their strengths and weaknesses [36]. The aforementioned studies on CVCARSs employ mainly simulation-based optimization methods for given fixed process configurations. Despite a vast reference on this matter, there are no studies on the simultaneous optimization of the process configuration, process-unit sizes, and operating conditions of CVCARSs running with low-grade waste heat, using mathematical programming techniques and rigorous optimization algorithms. The current work focuses on this aspect. 

This paper is a logic continuation of the work recently published by Mussati et al. [11], where an optimization model for the conventional series flow double-effect H_2_O-LiBr VARS was presented. The model is extended to a superstructure-based model to embed several candidate configurations with the aim to include the configuration of the process as an optimization variable. Then, the resulting VARS model is coupled to a model of the conventional VCRS, thus obtaining the desired model of the Vapor Compression-Absorption Cascade Refrigeration System (VCACRS). This model allows systematically determining the optimal configuration of the VCACRS from the proposed superstructure, the process-unit sizes, and the operating conditions simultaneously. The number of degrees of freedom of the resulting optimization model is significantly increased with respect to both standalone processes, thus allowing to find novel and/or improved system configurations.

## 2. Process Description 

### 2.1. Vapor Absorption Refrigeration System (VARS)

**Figure 1:**
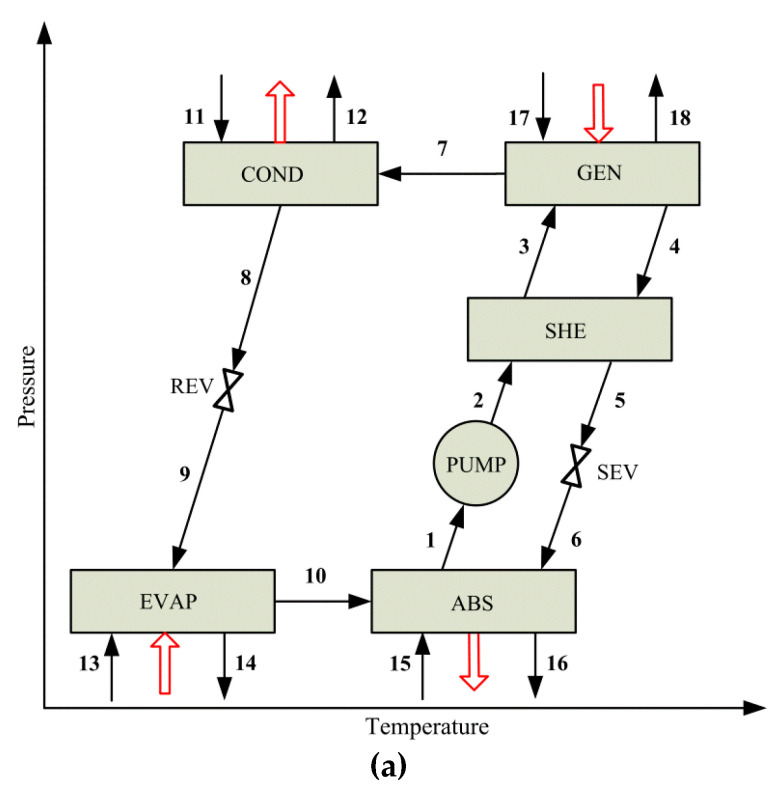

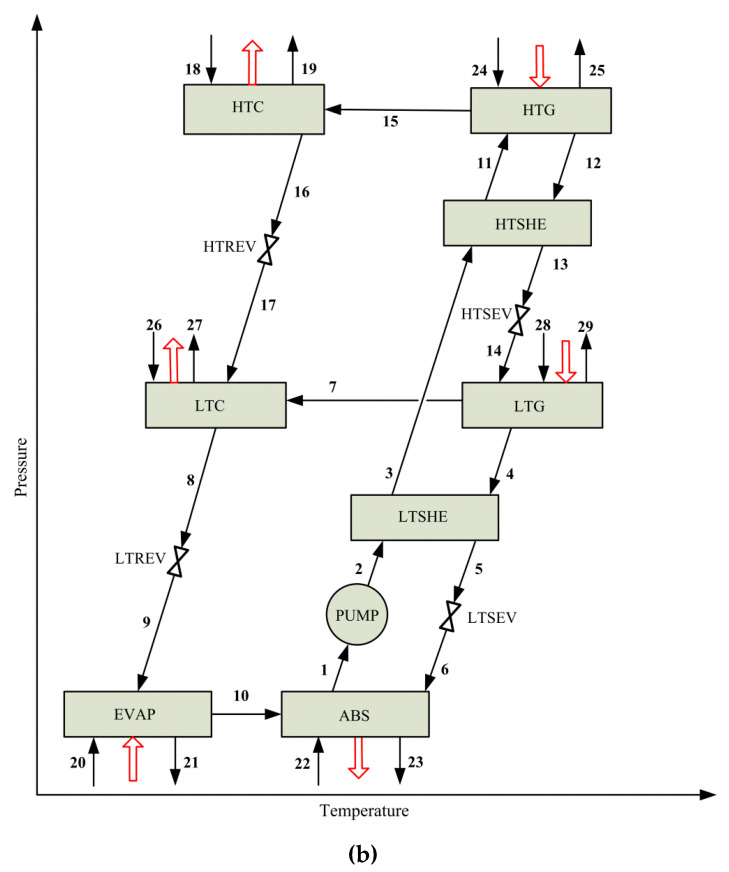
Schematics of vapor absorption refrigeration systems: (**a**) single-effect system; (**b**) series flow double-effect system.

Figure 1 shows the schematics of a single-effect and a series flow double-effect H_2_O-LiBr VARS. The double-effect system involves two generators G (the low-temperature generator LTG and the high-temperature generator HTG), two condensers C (LTC and HTC), and two LiBr solution heat exchangers SHE (LTSHE and HTSHE). Additionally, two solution expansion valves (LTSEV and HTSEV), two refrigerant expansion valves (LTREV and HTREV), a solution pump (PUMP), an absorber (ABS), and an evaporator (EVAP) are involved. 

The refrigeration process is taking place in EVAP. The refrigerant leaving EVAP is absorbed by the strong LiBr solution that enters ABS, producing a weak LiBr solution stream. The heat of the absorption process is removed by the cooling water. Compared to the energy input required in HTG and LTG, the electrical power required by PUMP to pump the LiBr solution is negligible. In LTSHE and HTSHE, the strong and weak LiBr solutions exchange heat resulting in a decrease of the heating utility demand in both LTG and HTG. In HTG and LTG, the refrigerant (H_2_O) is separated from the corresponding weak LiBr solution obtaining a strong LiBr solution stream and a vapor stream in each. As the solute (LiBr) determines an increase of the boiling point of the solution with respect to that of the refrigerant (H_2_O), the separated vapor in both generators is at superheated conditions. Then, the vaporized refrigerant streams generated in HTG and LTG are condensed in HTC and LTC, respectively, using cooling water. The operating pressure in EVAP is achieved by means of LTREV.

### 2.2. Vapor Compression Refrigeration System (VCRS)

**Figure 2:**
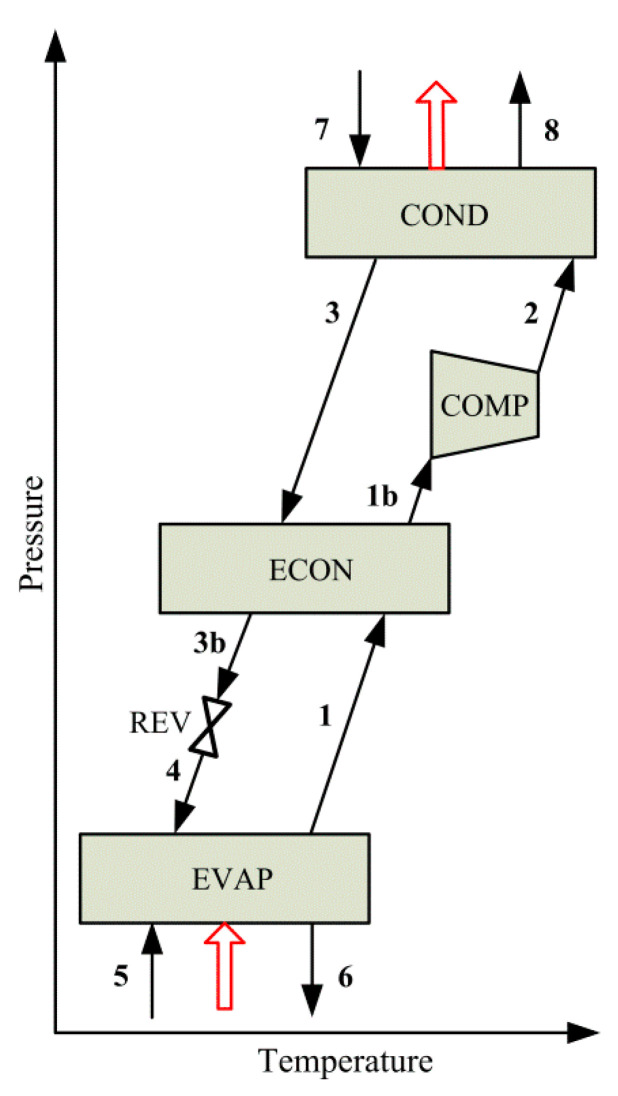
Schematic of a simple vapor compression refrigeration system.

Unlike VARSs, VCRSs operate with electrical power as driving energy. Figure 2 shows a schematic of a simple VCRS. The system consists of an evaporator (EVAP), a compressor (COMP), a condenser (COND), a refrigerant expansion valve (REV), and an economizer (ECON).

By comparing Figure 1a and Figure 2, it can be seen that the compressor in a simple VCRS replaces the absorber, the pump, the solution heat exchanger, the generator, and the expansion valve involved in a conventional single-effect VARS.

## 3. Problem Statement

**Figure 3:**
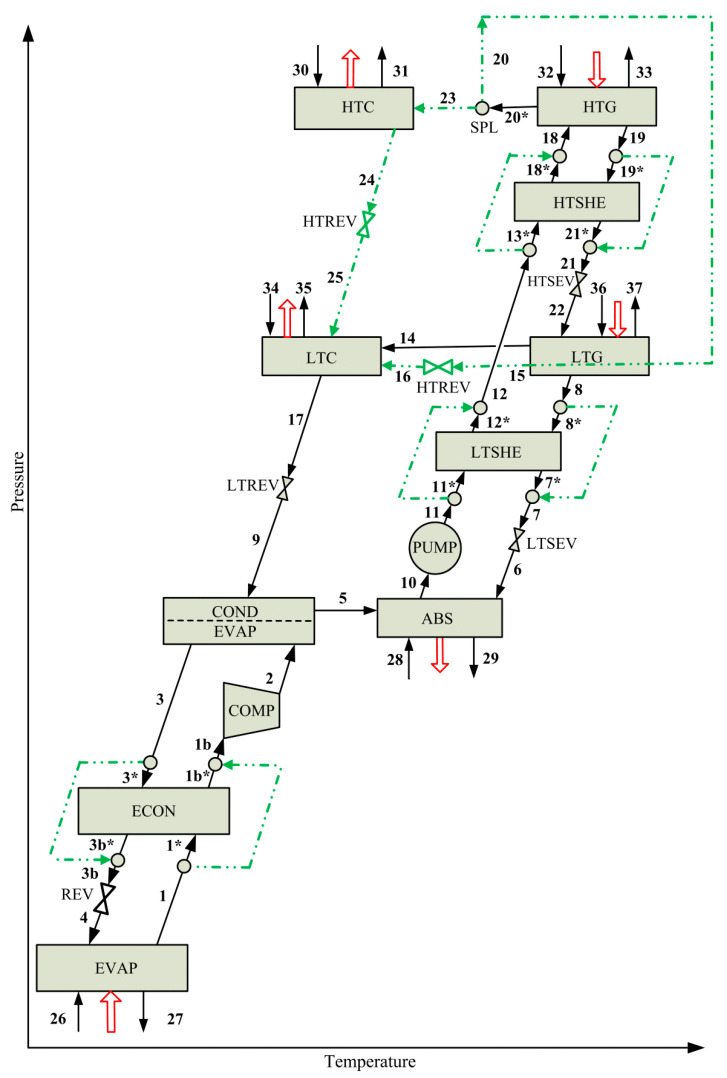
Schematic of the superstructure of the vapor compression-absorption cascade refrigeration system VCACRS, which is formed by a series flow double-effect H_2_O-LiBr VARS and a simple VCRS operated with R134a.

As mentioned in the Introduction section, the simultaneous optimization of VCACRSs by applying mathematical programming is addressed. Several possible process configurations are embedded in a single superstructure representation of the studied system (Figure 3), which is a combined process formed by a series flow double-effect H_2_O-LiBr VARS and a simple VCRS operating with R134a.

The proposed superstructure involves at least 10 alternative configurations, which differ in the way the components HTC, LTC, HTG, LTG, HTSHE, and LTSHE are combined or interconnected, or if some of them (HTC, HTSHE, LTSHE, and ECON) are removed from a given configuration. The components of the compression cycle (EVAP, COMP, COND/EVAP, REV) and ABS, PUMP, LTG, HTG, LTC, LTREV, LTSEV, and HTSEV of the absorption cycle are fixed in the superstructure i.e., they are present in all configurations. For instance, a candidate configuration may include HTC, HTSHE, and LTSHE; other candidate configuration may integrate energetically HTCG with LTG through the splitter SPL with elimination of HTC, but keeping HTSHE and LTSHE; other options may integrate energetically HTG and LTG through SPL with elimination of HTC as well as LTSHE and/or HTSHE from the superstructure, among other alternatives.

The optimization problem can be stated as follows. Given are (a) the superstructure of VCACRS (Figure 3) that embeds a number of combinations of the aforementioned optional and fixed system components, (b) specified values of evaporator temperature and refrigeration capacity of −17.0 °C and 50.00 kW, respectively, and (c) steam at 130.0 °C and cooling water at 25.0 °C as utilities. The problem consists on finding the optimal VCACRS configuration and the sizes and operating conditions of all system components that minimize the total heat transfer area (THTA) of VCACRS while satisfying the mentioned design specifications. 

The obtained optimal solution is compared in detail to a design reported in Colorado and Rivera [31], which is used as a reference design for this paper.

## 4. Modeling

### 4.1. Process Model

The mathematical model includes the mass and energy balances for each system component and the calculation of the corresponding heat transfer areas and driving forces. The considered complete mathematical model of VCACRS combines a model of double-effect H_2_O-LiBr VARS [11] and a model of a simple R134a VCRS.

#### 4.1.1. Definitions

Let SC be the set of the system components k:

(1) $$\mathrm{SC}=\left\{\begin{array}{l} \mathrm{EVAP},\;\mathrm{COMP},\;\mathrm{COND}/\mathrm{EVAP},\;\mathrm{REV},\;\mathrm{ECON},\;\mathrm{ABS},\;\mathrm{PUMP},\\ \mathrm{LTG},\;\mathrm{HTG},\;\mathrm{LTC},\;\mathrm{LTREV},\;\mathrm{LTSEV},\;\mathrm{HTSEV} \end{array}\right\}$$

Let SS represent the set of all system streams i and IN_k_ and OUT_k_ the sets of the system streams i entering and leaving a system component k, respectively. 

Let M, X, Q, W, and H be the mass flow rate (kg∙s^−1^), mass fraction (kg∙kg^−1^), heat load (kW), power (kW), and enthalpy flow rate (kW).

#### 4.1.2. Steady-State Balances for the k-th System Component

- Total mass balance:
  (2) $$\sum_{i\in {\mathrm{IN}}_{k}}M_{i,k}-\sum_{i\in {\mathrm{OUT}}_{k}}M_{i,k}=0,\;\forall k\in \mathrm{SC}$$
- Mass balance of component j = LiBr:
  (3) $$\sum_{i\in {\mathrm{IN}}_{k}}M_{i,k}\cdot X_{j,i,k}-\sum_{i\in {\mathrm{OUT}}_{k}}M_{i,k}\cdot X_{j,i,k}=0,\;\forall k\in \mathrm{SC},\;j=\mathrm{LiBr}$$
- Energy balance (with negligible potential and kinetic energy changes):
  (4) $$\begin{array}{l} Q_{u,k}-W_{k}+\sum_{i\in {\mathrm{IN}}_{k}}H_{i,k}-\sum_{i\in {\mathrm{OUT}}_{k}}H_{i,k}=0,\;\forall k\in \mathrm{SC}\\ Q_{u,k}=\pm (H_{u,\mathrm{in},k}-H_{u,\mathrm{out},k}) \end{array}$$

#### 4.1.3. Design Constraints

- Heat transfer area of a system component k (HTA_k_):
  (5) $$Q_{k}=U_{k}\cdot {\mathrm{HTA}}_{k}\cdot {\mathrm{LMTD}}_{k},\;\forall k\in \mathrm{SC}$$
  where LMTD_k_ is the logarithmic mean temperature difference, which is calculated as:
  (6) $${\mathrm{LMTD}}_{k}=\frac{\Delta T_{k}^{H}-\Delta T_{k}^{C}}{\ln\frac{\Delta T_{k}^{H}}{\Delta T_{k}^{C}}},\;\forall k\in \mathrm{SC}$$
  $\Delta T_{k}^{H}$ and $\Delta T_{k}^{C}$ are the temperature differences at the hot and cold sides, respectively.
- Total heat transfer area of VCACRS (THTA):
  (7) $$\mathrm{THTA}=\sum_{k\;}{\mathrm{HTA}}_{k},\;\forall k\;\in \mathrm{SC}$$
- Heat exchanger effectiveness factor (ε):
  The effectiveness factor ε of the solution LTSHE (Equation (8)) and HTSHE (Equation (9)) is based on the strong solution side:
  (8) $$\varepsilon_{\mathrm{LTSHE}}=\frac{M_{11*}\cdot X_{11*}\cdot \left(T_{12*}-T_{11*}\right)}{M_{8*}\cdot X_{8*}\cdot \left(T_{8*}-T_{11*}\right)}$$
  (9) $$\varepsilon_{\mathrm{HTSHE}}=\frac{M_{13*}\cdot X_{13*}\cdot \left(T_{18*}-T_{13*}\right)}{M_{19*}\cdot X_{19*}\cdot \left(T_{19*}-T_{13*}\right)}$$
- Inequality constraints on stream temperatures:
  Inequality constraints are added to avoid temperature crosses in the system components. For instance, Equations (10) and (11) are considered for LTC, where δ is a small (positive) value (in this case δ = 0.1). Similar inequality constraints are considered for the remaining system components.
  (10) $$T_{35}\geq T_{14}+\delta$$
  (11) $$T_{34}\geq T_{17}+\delta$$
- Other modeling considerations:
  The model also includes the mass balance corresponding to the splitter SPL (Equation (12)), which allows to optionally consider the heat integration between HTG and LTG in some candidate configurations.
  (12) $$M_{20*}=M_{20}+M_{23}$$

According to Equation (12), if M_23_ = 0, then this implies that HTC is removed and, consequently, the energy contained in stream 20* is transferred in LTG through stream 20.

The elimination (or selection) of LTSHE and HTSHE can be directly dealt with the values of their effectiveness factors (ε_LTSHE_ and ε_HTSHE_, respectively), Equations (8) and (9).

According to Equation (8), if T_12*_ = T_11*_ (no heat transfer), then η_LTSHE_ = 0. In analogy, in Equation (9), if T_18*_ = T_13*_ (no heat transfer), then η_HTSHE_ = 0. From this analysis, it can be concluded that the consideration of both η_LTSHE_ and η_HTSHE_ as optimization variables with proper lower and upper bounds (1 × 10^−3^% and 99.0%, respectively) makes it unnecessary to propose bypass streams (as shown in Figure 3) to eliminate LTSHE and HTSHE from a given configuration. The mathematical formulation of bypass streams would require the inclusion of binary decision variables and, consequently, the transformation of the NLP model into a MINLP model.

In summary, Equations (8), (9) and (12) allow that the candidate process configurations to be embedded and contemplated simultaneously in the mathematical model, whose solution provides the optimal one.

### 4.2. Objective Function

The objective function is the minimization of the total heat transfer area of the system (THTA) to obtain a evaporator temperature and refrigeration capacity of −17.0 °C and 50.00 kW, respectively, using steam at 130.0 °C and cooling water at 25.0 °C as utilities. The optimization problem is formulated as follows:

(13) $$\begin{array}{l} \mathrm{Min}\;\left(\mathrm{THAT}\right)\\ s.t.\\ \left\{ \begin{array}{l} \mathrm{Eqs}.\;(1)–\left(12\right)\\ \mathrm{Property}\;\mathrm{estimation}\;\mathrm{expressions}\\ Q_{\mathrm{EVAP}}=50.00\;\mathrm{kW}\\ T_{\mathrm{EVAP}}=-17.0\;{}^{\circ}C\\ T_{\mathrm{steam}}=130.0\;{}^{\circ}C,\;T_{\mathrm{cooling}\;\mathrm{water}}=25.0\;{}^{\circ}C \end{array}\right. \end{array}$$

## 5. Results and Discussion

The optimization model was implemented in the platform GAMS v.23.6.5 [37] and solved with the solver CONOPT 3 v.3.14W [38].

### 5.1. Model Verification

Before solving the optimization problem stated in the previous section, the proposed model was succesfully verified using the data reported by Colorado and Rivera [31], which is here used as a reference case (referred to as the Colorado and Rivera’s solution ‘CRS’). To this end, it was necessary to set certain numerical values to consider the same configuration and operating conditions as in [31]. Table 1 and Table 2 compare the model’s output results with the solution reported in [31] for the analyzed system operating with R134a in VCRS and H_2_O-LiBr in VARS. The values that were fixed are marked with (^a^) in these tables. 

From the comparison of values presented in Table 1 and Table 2, it can be concluded that the results obtained with the implemented model is in agreement with the data reported in [31].

### 5.2. Optimization Results

Table 3 lists the main model parameters with the numerical values.

**Figure 4:**
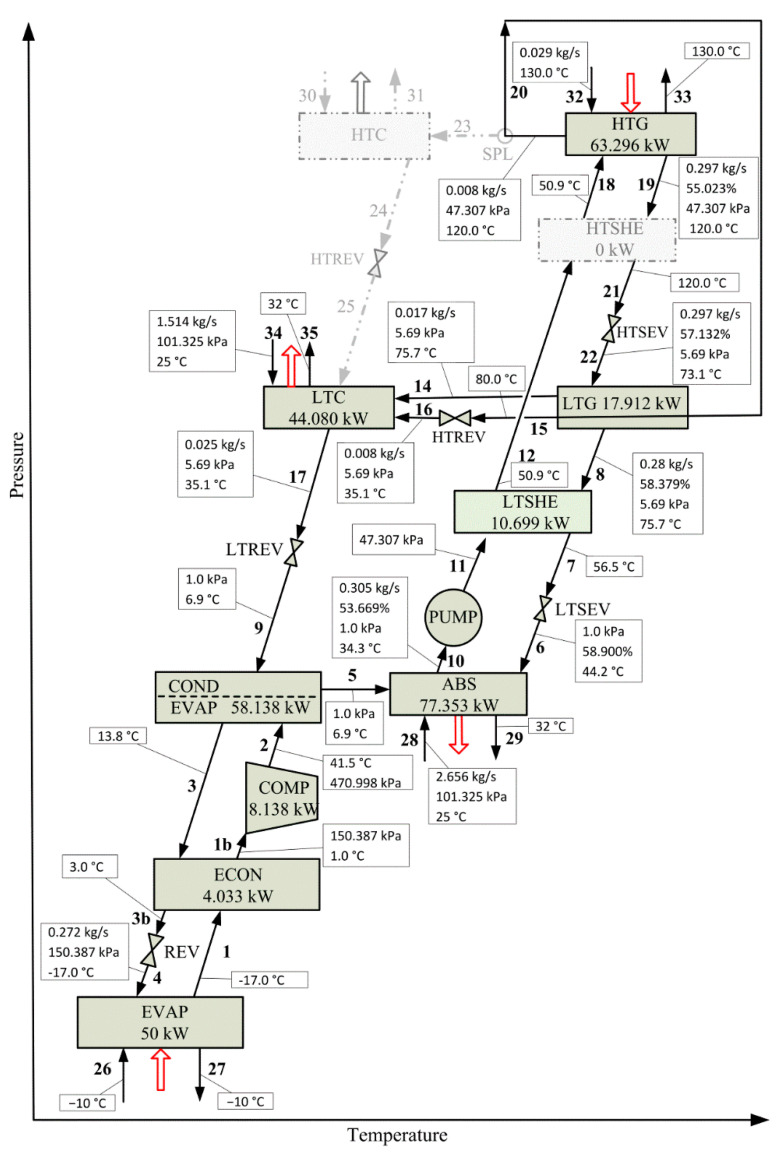
Optimal solution OS. Process configuration resulting from the superstructure proposed in Figure 3 by minimizing the total heat transfer area (THTA) for a refrigeration capacity of 50.00 kW.

Figure 4 illustrates the optimal configuration selected from the proposed superstructure and the properties of the process streams entering and leaving each process component, and Table 4 reports the optimal values of the heat transfer area, heat load, and driving force of each system component. This optimal solution is hereafter referred to as ‘OS’. As shown in Figure 4, the components HTC and HTSHE were removed from the proposed superstructure. No fraction of refrigerant separated in HTG is send to HTC, i.e., the refrigerant is completely used in LTG as the heating medium, where no (external) heating utility is required. Regarding HTSHE, the optimal value of its effectiveness factor ε_HTSHE_ is 6.6 × 10^−28^ because the temperature difference of the strong LiBr solution streams 19 and 21 at the inlet and outlet of HTSHE, respectively, is practically null. However, LTSHE is selected in the optimal solution with an optimal ε_LTSHE_ value of 40.3%. The temperature difference of the strong LiBr solution streams 8 and 7 at the inlet and outlet of LTSHE, respectively, is 19.2 K, and that of the weak solution streams 12 and 11 at the outlet and inlet of LTSHE, respectively, is 16.6 K.

**Figure 5:**
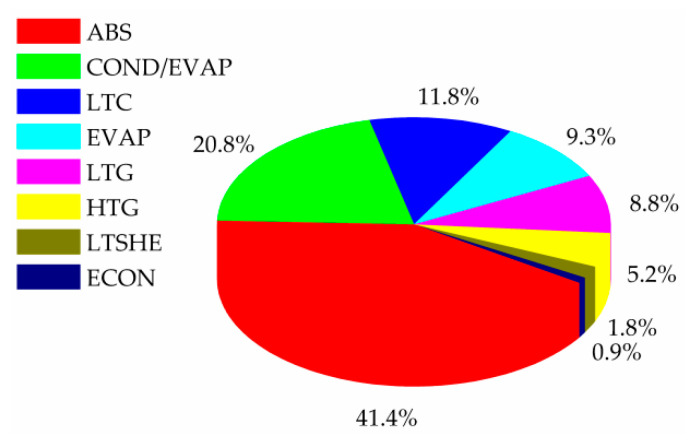
Optimal solution OS. Distribution of the total heat transfer area (THTA) among the system components obtained by minimizing THTA, for a refrigeration capacity of 50.00 kW.

This obtained configuration is different from the configurations reported so far in the literature for combinations of double-effect H_2_O-LiBr VAR and VCR systems. According to Table 4, it requires a minimal THTA value of 24.980 m^2^, which is optimally distributed among the process units as is indicated in Figure 5.

The component ABS requires the largest heat transfer area (10.339 m^2^), which represents 41.4% of THTA, followed by COND/EVAP, which allowed coupling the two refrigeration systems, representing 20.8% (5.225 m^2^) of THTA. The components LTC, EVAP, and LTG require similar heat transfer areas (2.959, 2.331, and 2.190 m^2^) contributing with 11.8%, 9.3%, and 8.8% of THTA, respectively. The heat transfer area required in LTSHE is higher than twice the required in ECON (0.457 m^2^ vs. 0.191 m^2^). The optimal LiBr concentration values of the strong solutions at LTG and HTG are 58.379% and 55.023%, respectively, while the corresponding to the weak solution at ABS is 53.669%. The optimal operating pressures at LTG and HTG are 5.69 and 47.307 kPa, respectively. With regard to heating utility, which is provided by steam, the system requires a mass flow rate of 0.029 kg∙s^−1^ at 130.00 °C. Regarding the cooling utility, which is provided by water, the system requires a mass flow rate of 1.514 kg∙s^−1^ in LTC and 2.656 kg∙s^−1^ in ABS, with inlet and outlet temperatures of 25.0 and 32.0 °C, respectively. The compressor COMP requires an amount of electrical power of 8.138 kW. The specified refrigeration capacity of 50.00 kW is obtained by evaporating 0.272 kg∙s^−1^ of R134a at 150.390 kPa and −17.0 °C. The refrigerant R134a of the VCRS transfers heat to the refrigerant H_2_O of the VARS in COND/EVAP at a heat flow rate of 58.138 kW. The total mass flow rate of refrigerant H_2_O evaporated in the VARS cycle is 0.025 kg∙s^−1^, of which 8.0 × 10^−3^ kg∙s^−1^ is obtained in HTG and 0.017 kg∙s^−1^ in LTG.

Compared to the reference case CRS (Section 5.1), the THTA value decreased 16.7% (5.015 m^2^, from 30.000 to 24.980 m^2^) implying an increase of 18.19 kW in the heat load in HTG and a decrease of 1.33 kW in COMP. The obtained values determine a COP value of 0.700, which is 0.216 less than that corresponding to CRS (0.916). Then, it is interesting to solve the same optimization problem, i.e., to minimize THTA but now considering the COP value estimated for CRS. This optimal solution is hereafter referred to as ‘SubOS’, which is a suboptimal solution with respect to the optimal solution OS.

#### Comparison between the Optimal Solution SubOS and the Reference Case CRS

This section presents a comparison between the optimal solution obtained by the superstructure-based model and that corresponding to the reference case CRS [31]. To this end, the optimization model is solved for the same values of refrigeration capacity (50.00 kW), heat load in HTG (45.10 kW) and mechanical power required by the compressor (9.464 kW), as considered in the reference case. 

**Figure 6:**
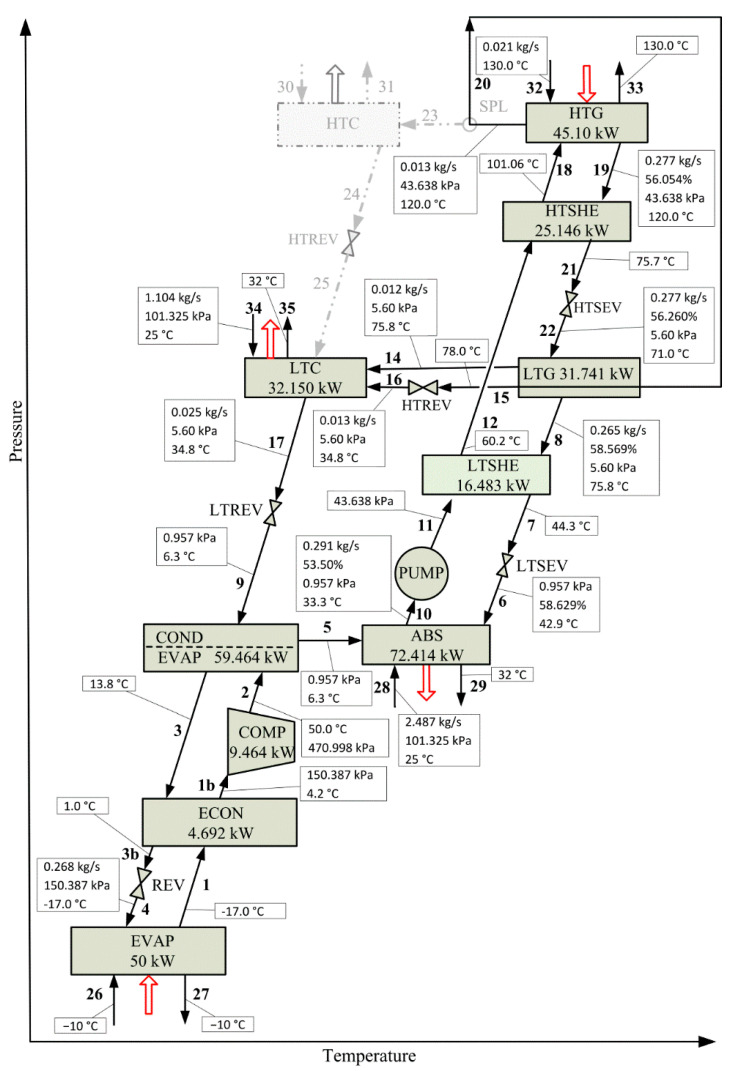
Reference case CRS. Process configuration and operating conditions reported in [31] for a specified refrigeration capacity of 50.00 kW, a heat load of 45.10 kW in HTG, and a mechanical power of 9.464 kW in COMP.

**Figure 7:**
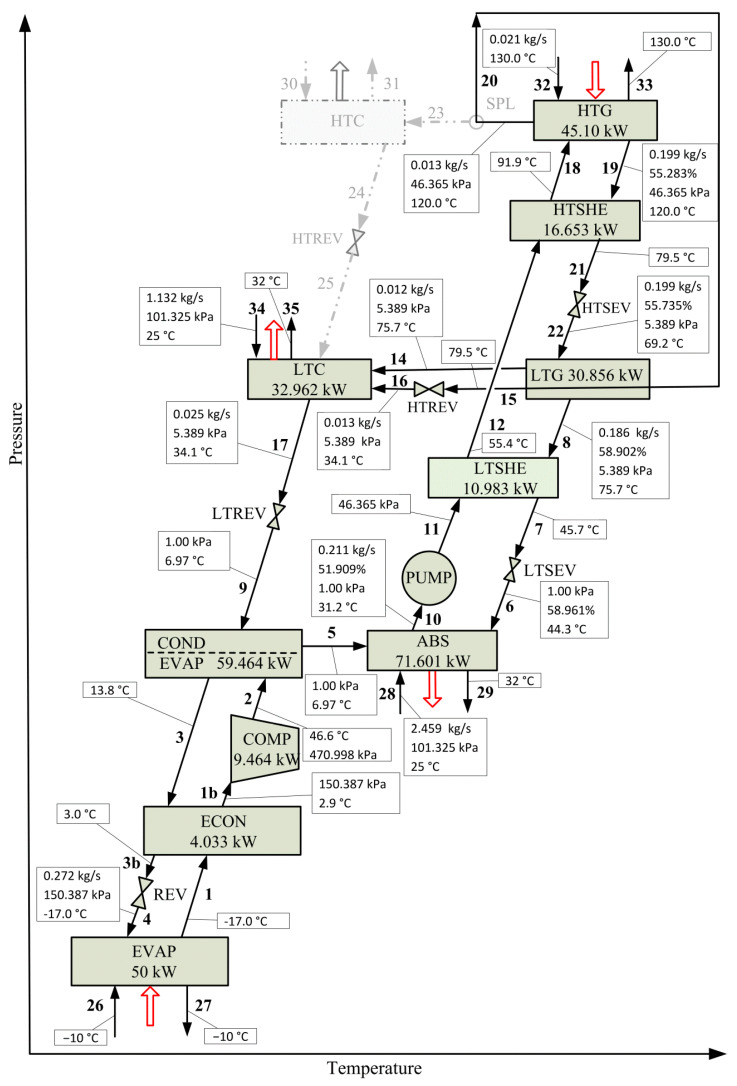
Optimal solution SubOS. Process configuration and operating conditions obtained for a refrigeration capacity of 50.00 kW, a heat load of 45.10 kW in HTG, and a mechanical power of 9.464 kW in COMP.

Figure 6 and Figure 7 present the process configurations and the operating conditions of each system component corresponding to CRS and SubOS, respectively. Table 5 compares the values of the heat load Q, heat transfer area HTA and driving force DF of each system component and the total heat transfer area THTA between CRS and SubOS cases. 

For the same input energy in HTG and mechanical power in COMP used in CRS, a main result to highlight is that HTSHEX is now selected by SubOS and THTA decreases with respect to CRS. From Table 5, it can be observed several changes in the operating conditions of the system components compared to CRS. The THTA required in SubOS is 7.3% smaller than that required in CRS (27.824 m^2^ vs. 30.000 m^2^). Despite the fact that COND/EVAP, ABS, and LTC increase their heat transfer areas compared to CRS (in total 1.543 m^2^, from 17.881 m^2^ to 19.422 m^2^), the remaining system components HTG, LTG, COND, ECON, LSHEX, and HSHEX decrease their heat transfer areas (in total 3.719 m^2^, from 9.789 m^2^ to 6.070 m^2^), thus resulting in a net decrease of 2.176 m^2^. For instance, although the heat load in COND/EVAP is the same in both configurations (59.463 kW), the respective heat transfer area required in SubOS is 5.6% higher than that required in CRS (11.438 m^2^ vs. 10.828 m^2^) because the associated driving force in SubOS is smaller than that in CRS (18.5 K vs. 20.3 K for the subcooling process and 6.8 K vs. 7.5 K for the condensation process, as shown in Table 5). The differences in the driving force values in both solutions is because of the different inlet temperatures of the refrigerant R134a in the COND/EVAP. The operating temperatures are 46.6 and 50.0 °C in SubOS and CRS, respectively, while the same operating pressures are considered in both solutions (470.998 kPa in COND/EVAP and 150.387 in EVAP). However, it is necessary to increase the refrigerant flow in SubOS by 0.004 kg·s^−1^ (from 0.268 to 0.272 kg·s^−1^) to provide the mechanical power required in COMP (9.464 kW). As the temperature leaving REV in SubOS is 2.0 °C higher than CRS, the vapor quality of the stream entering EVAP valve increases by 0.013 (from 0.113 in CRS to 0.126 in SubOp) in order to maintain both the isoenthalpic condition in REV and the specified refrigeration capacity (50.00 kW). 

The heat load in ABS in SubOS is lower than that in CRS (71.601 kW vs. 72.414 kW) but the required area is larger (11.438 m^2^ vs. 10.828 m^2^) because the driving force in SubOS is lower than that in CRS (8.9 K vs. 9.5 K). A different behavior is observed for LTG between the heat load, heat transfer area, and driving force. The heat load in LTG in SubOS is 0.885 kW lower than that in CRS (30.856 kW vs. 31.741 kW) and the heat transfer area is 1.956 m^2^ lower (3.164 m^2^ vs. 5.120 m^2^) because the driving force in SubOS is 2.4 K higher than that in CRS (6.5 K vs. 4.1 K). This behavior is also observed for HTG. With respect to the solution heat exchangers, the total heat exchanged in HTSHE and LTSHE in CRS is 13.993 kW higher than that in SubOS (41.629 kW vs. 27.636 kW), requiring 1.433 m^2^ more of heat transfer area (2.710 m^2^ vs. 1.277 m^2^).

Regarding the LiBr solution concentration values, SubOS shows values lower than CRS, with the particularity that the concentration difference between the weak and strong solutions at HTG in SubOS is higher than that in CRS (3.374% vs. 2.554%). The LiBr solution concentration values leaving the LTG are almost similar in both solutions (58.569% in CRS and 58.902% in SubOS). 

An important result to note is that the total mass flow rates of H_2_O refrigerant circulating in the absorption subsystem are the same in both solutions. However, the flowrates of the weak and strong LiBr solution in SubOS are lower than those in CRS (weak: 0.212 vs. 0.291 kg·s^−1^; strong: 0.199 vs. 0.277 kg·s^−1^). While the operating pressure at HTG in SubOS is higher than that in CRS (46.365 kPa vs. 43.638 kPa) and it is almost similar at ABS (0.957 kPa in CRS and 1.000 kPa in SubOS) and LTG (5.600 kPa in CRS and 5.389 kPa in SubOS). Although the total flowrate of cooling water required in ABS and COND is the same in both solutions (3.591 kg·s^−1^)—as the heat loads at HTG and EVAP, mechanical power at COMP, and inlet and oultet temperatures of the cooling water are the same—the individual cooling requiriments in ABS and COND are different in both solutions. In SubOS, COND and ABS require 1.132 kg·s^−1^ and 2.459 kg·s^−1^ of cooling water, respectively; while, in CRS, they require 1.104 kg·s^−1^ y 2.487 kg·s^−1^, respectively.

As a summary of the comparative analysis between both configurations, it can be concluded that the obtained SubOS solution is preferred over the reported CRS solution as the former requires less THTA for the same requirements of heating and cooling utilities (steam and cooling water, respectively), thus implying a lower total annual cost (investment plus operating costs).

**Figure 8:**
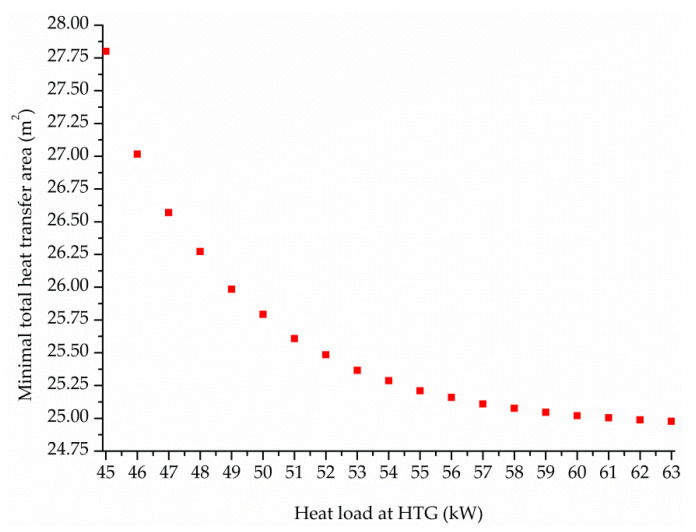
Minimal THTA vs. heat load at HTG for a refrigeration capacity of 50.00 kW.

**Figure 9:**
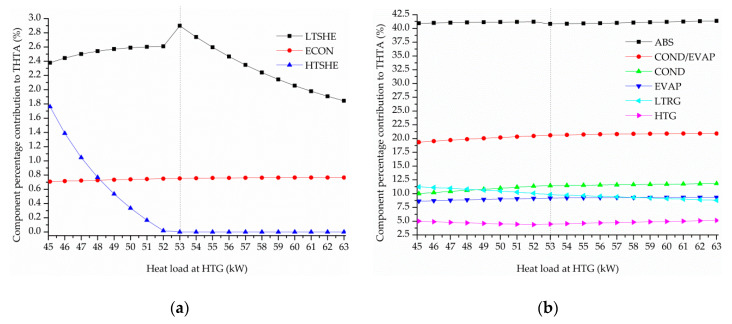
Optimal percentage contribution of each component to THTA vs. heat load at HTG, for a refrigeration capacity of 50.00 kW. (**a**) LTSHEX, ECON, and HTSHEX, (**b**) ABS, COND/EVAP, COND, EVAP, LTG, and HTG.

Finally, the influence of the heat load at HTG on the selection or elimination of the HTSHE and on the total heat transfer area THTA is investigated by keeping a refrigeration capacity of 50.00 kW, in order to identify the input energy level that determines the elimintation of HTSHE from the optimal solutions. To this end, the heat load at HTG is parametrically varied from 45.10 to 63.00 kW and the mathematical optimization model is solved to find the minimal THTA value for each case. Figure 8 plots the minimal THTA vs. the heat load at HTG and Figure 9 shows the optimal percentage contribucion of each system component to THTA. 

As expected, the higher the heat load at HTG the lower the THTA (Figure 8). Regarding the selection or elimination of HTSHE from the process configuration, Figure 9a shows that HTSHE is included in the optimal solutions for HTG heat load values in the variation range between 45.00 and 52.00 kW and that it is removed from the optimal solutions for a HTG heat load value equal or higher than 53.00 kW. 

## 6. Conclusions

Superstructure-based optimization of a vapor compression-absorption cascade refrigeration system consisting of a series flow double-effect H_2_O-LiBr absorption system and an R134a compression system, which embeds several candidate process configurations to consider the configuration as an optimization variable, was successfully addressed by applying nonlinear mathematical programming. 

As a main result, a novel configuration of the combined process not previously reported in the literature—according to the best of our knowledge—was obtained when the total heat transfer area of the system was minimized. Two characteristics of the resulting optimal configuration are (a) the elimination of the high-temperature LiBr solution heat exchanger HTSHE; and (b) the energy integration between the high-temperature generator HTG and the low-temperature generator LTG, thus eliminating the presence of the (separated) high-temperature condenser HTC, i.e., no fraction of the refrigerant separated in HTG is sent to HTC since it is totally used in LTG as the heating medium, where no external heating utility is required to produce extra vapor at low temperature. 

From a quantitative point of view, the component ABS shows the largest heat transfer area, which represents around 41% of the total heat transfer area. It is followed by COND/EVAP, which allowed coupling the two refrigeration systems by evaporating refrigerant H_2_O in the absorption cycle while condensing R134a in the compression cycle, which represents around 20% of the total heat transfer area.

Additionally, the obtained optimal solution was compared with the solution corresponding to a base configuration recently reported in the literature—used as a reference design—for the same coefficient of performance (COP), working fluids, refrigeration capacity and evaporator temperature (50.00 kW and −17.0 °C, respectively). The comparison showed that the obtained minimal total heat transfer area is around 7.3% smaller than the required in the reference case. 

Finally, the influence of the heat load at HTG on the total heat transfer area THTA and the selection or elimination of HTSHE for a same refrigeration capacity of 50 kW and a evaporator temperature of −17.0 °C was also investigated. The HTG heat load was parametrically varied from 45.0 to 63.0 kW. It was found that HTSHE is included in the optimal solutions for HTG heat load values in the variation range between 45.00 and 52.00 kW and that it is removed from the optimal solutions for a HTG heat load value equal or higher than 53.00 kW. The component HTC is always eliminated in the obtained optimal solutions.

## Figures and Tables

**Table 1:** Model validation. Comparison of mass flow rates (M), temperatures (T), pressures (P), and LiBr solution concentrations (X) of some representative process streams between the model outputs and reported data [31]. (T_EV__AP_ = −17.0 °C, T_HTG_ = 120.0 °C, Q_EVAP_ = 50.00 kW).

	M (kg∙s^−1^)		T (°C)		P (kPa)		X (% p/p)	
Stream	Ref. [31]	This Work	Ref. [31]	This Work	Ref. [31]	This Work	Ref. [31]	This Work
1	0.267	0.268	−17.0	−17.0 ^a^	150.8	150.387	–	–
1b	0.267	0.268	4.7	4.4	150.8	150.387	–	–
2	0.267	0.268	49.9	50.1	472.9	470.998	–	–
3	0.267	0.268	14.0	14.0 ^a^	472.9	470.998	–	–
3b	0.267	0.268	1.0	1.0 ^a^	472.9	470.998	–	–
5	0.025	0.025	7.0	6.4	1.0	0.957	–	–
6	0.266	0.255	47.6	44.4	1.0	0.957	58.9	58.569
8	0.266	0.255	77.1	76.4	5.6	5.600 ^a^	58.9	58.569
10	0.291	0.280	35.0	34.1	1.0	0.958	53.8	53.498
14	0.011	0.011 ^a^	77.1	76.4	5.6	5.600 ^a^	–	–
18	0.291	0.280	99.0	102.3	42.1	43.638	53.8	53.498
19	0.278	0.266	120.0	120.0 ^a^	42.1	43.638	56.3	56.054

^a^ Fixed value.

**Table 2:** Model validation. Comparison of heat loads (Q), compressor work (W), and coefficients of performance (COP) between the model outputs and reported data [31] (T_EVAP_ = −17.0 °C, T_HTG_ = 120.0 °C, Q_EVAP_ = 50.00 kW).

Item	Ref. [31]	This Work
Heat load (kW)		
– High-temperature generator, HTG	45.80	45.10
– Absorber, ABS	72.63	72.414
– Condenser, COND	32.27	32.150
– Evaporator, EVAP	50.00	50.00 ^a^
Work (kW)		
– Compressor, COMP	9.10	9.464
COP (dimensionless)		
– VARS cycle	1.29	1.312
– VCRS cycle	5.49	5.283
– VCRS-VARS cascade cycle	0.91	0.916

^a^ Fixed value.

**Table 3:** Numerical values of the model parameters.

Parameter	Value
Cooling capacity (kW)	50.00
Utility inlet/outlet temperature (°C):	
– Cooling water in condensers and absorbers	25.0/32.0
– Steam in generators	130.0
Overall heat transfer coefficient (kW∙m^−2^∙°C^−1^):	
– Evaporator, U_EVAP_	1.50
– Absorber, U_ABS_	0.70
– Condenser, U_COND_	2.50
– Generator, U_GEN_	1.50
– Cascade condenser	0.55
– Solution heat exchanger, U_SHE_	1.00

**Table 4:** Optimal solution OS. Heat loads (Q), heat transfer areas (HTA), and logarithmic mean temperature differences (LMTD) obtained by minimizing the total heat transfer area (THTA) for a cooling capacity of 50.00 kW.

Component	HTA (m^2^)	Q (kW)	LMTD (K)
ABS	10.339	77.353	10.7
COND/EVAP	0.277 ^a^/4.948 ^b^ 5.225 ^c^	7.068 ^a^/51.070 ^b^ 58.138 ^c^	16.9 ^a^/6.9 ^b^
LTC	2.959	44.080	6.0
EVAP	2.331	50.00	7.1
LTG	2.190	17.912	5.4
HTG	1.288	63.296	32.7
LTSHE	0.457	10.699 (ε = 40.3%)	23.4
ECON	0.191	4.033	16.2
HTSHE	1.53 × 10^−24^	1.060 × 10^−22^ (ε = 0)	69.0
HTC	–	–	–
Total	24.980 (THTA)		

^a^ Subcooling process; ^b^ condensation process; ^c^ total.

**Table 5:** Comparison of heat load (Q), heat transfer area (HTA), and driving force (DF) values between the reference case (CRS) [31] and the optimal solution obtained in this work (SubOS), for a refrigeration capacity of 50.00 kW.

	Ref. [31]			SubOS (This Work)		
Component	Q (kW)	HTA (m^2^)	DF (K)	Q (kW)	HTA (m^2^)	DF (K)
EVAP	50.00	2.331	7.150	50.00	2.331	7.150
COND/EVAP	9.127 ^a^/50.336 ^b^ 59.463 ^c^	0.299 ^a^/4.469 ^b^ 4.768 ^c^	20.3 ^a^/7.5 ^b^	8.396 ^a^/51.068 ^b^ 59.464 ^c^	0.302 ^a^/4.948 ^b^ 5.25 ^c^	18.5 ^a^/6.8 ^b^
ABS	72.414	10.828	9.5	71.601	11.438	8.9
LTSHE	16.483	1.247	13.2	10.983 (ε = 54.348%)	0.637	17.2
LTG	31.741	5.120	4.1	30.856	3.164	6.5
HTSHE	25.146	1.463	17.2	16.653 (ε = 56.542%)	0.64	26.0
HTG	45.10	1.690	17.8	45.100	1.438	20.9
LTC	32.150	2.285	5.629	32.962	2.735	4.8
ECON	4.692	0.269	13.4	4.033	0.191	16.2
HTC	-	-	-	–	–	–
Total		30.000 (THTA)			27.824 (THTA)	

^a^ Subcooling process; ^b^ condensation process; ^c^ total.
